# Entanglement entropy and hyperuniformity of Ginibre and Weyl–Heisenberg ensembles

**DOI:** 10.1007/s11005-023-01674-y

**Published:** 2023-05-12

**Authors:** Luís Daniel Abreu

**Affiliations:** NuHAG, Faculty of Mathematics, University of Vienna, Oskar-Morgenstern-Platz 1, 1090 Vienna, Austria

**Keywords:** Determinantal point processes, Entanglement entropy, Weyl–Heisenberg ensembles, Hyperuniformity, 82D03, 58J50, 42

## Abstract

We show that, for a class of planar determinantal point processes (DPP) ${\mathcal {X}}$, the growth of the entanglement entropy $S({\mathcal {X}}(\Omega ))$ of $${\mathcal {X}}\ $$ on a compact region $\Omega \subset {\mathbb {R}}^{2d}$, is related to the variance ${\mathbb {V}}\left( {\mathcal {X}}(\Omega )\right)$ as follows:

$$\begin{aligned} {\mathbb {V}}\left( {\mathcal {X}}(\Omega )\right) \lesssim S\left( \mathcal {X(} \Omega \mathcal {)}\right) \lesssim {\mathbb {V}}\left( {\mathcal {X}}(\Omega )\right) . \end{aligned}$$

Therefore, such DPPs satisfy an *area law* $S\left( {\mathcal {X}}_{g} \mathcal {(}\Omega \mathcal {)}\right) \lesssim \left| \partial {\Omega } \right|$, where $\partial {\Omega }$ is the boundary of $\Omega$ if they are of *Class I hyperuniformity *(${\mathbb {V}}\left( {\mathcal {X}} (\Omega )\right) \lesssim \left| \partial {\Omega }\right|$), while the *area law is violated* if they are of *Class II hyperuniformity *(as $\ L\rightarrow \infty$, ${\mathbb {V}}\left( {\mathcal {X}} (L\Omega )\right) \sim C_{\Omega }L^{d-1}\log L$). As a result, the entanglement entropy of Weyl–Heisenberg ensembles (a family of DPPs containing the Ginibre ensemble and Ginibre-type ensembles in higher Landau levels), satisfies an area law, as a consequence of its hyperuniformity.

## Introduction

If one considers a partition of a many-particle state in two subregions, *the entanglement entropy* measures the degree of entanglement between the two regions, which is given by the von Neumann entropy of the reduced state in one of the regions. Entanglement entropy is nowadays a widely studied quantity in many-particle interacting systems [7, 10–12, 16, 22]. In this note we interpret the definition of entanglement entropy for fermionic states given in [7, Proposition 7.2], in terms of planar determinantal point process (DPP) in ${\mathbb {R}}^{2d}$. This allows to define the entanglement entropy $S({\mathcal {X}}(\Omega ))$ of a DPP $${\mathcal {X}}\ $$in a compact subregion $\Omega \subset {\mathbb {R}}^{2d}$, as $S({\mathcal {X}}(\Omega ))= \textrm{trace}(f(T_{\Omega }))$, where $f(x)=-x\ln x-(1-x)\ln (1-x)$ and $T_{\Omega }$ is a Toeplitz operator defined with the correlation kernel of the DPP, with symbol the indicator function of $\Omega$ (see Sect. 2). Motivated by this definition, we will show that, for a class of planar DPPs for which $\textrm{trace}\left( T_{\Omega }^{p}\left( 1-T_{\Omega }\right) ^{p}\right)$ is bounded for $0<p<1$, which include the Ginibre ensemble and its higher Landau level versions [25], the following relations between the entanglement entropy $S({\mathcal {X}}(\Omega ))$ and the variance ${\mathbb {V}}\left( {\mathcal {X}}(\Omega )\right)$ hold:

1.1 $$\begin{aligned} {\mathbb {V}}\left( {\mathcal {X}}(\Omega )\right) \lesssim S\left( \mathcal {X(} \Omega \mathcal {)}\right) \lesssim {\mathbb {V}}\left( {\mathcal {X}}(\Omega )\right) \text {.} \end{aligned}$$

The entanglement entropy is said to satisfy an *area law* if $S\left( \mathcal {X(}\Omega \mathcal {)}\right) \lesssim \left| \partial {\Omega } \right|$, where $\left| \partial {\Omega }\right|$ is the measure of the perimeter of $\Omega$ or, asymptotically, for a dilated region $R{\Omega }$, if $\ {\mathbb {V}}\left( {\mathcal {X}}(\Omega \mathcal {)} \right) \sim R^{d-1}$ as $R\rightarrow \infty$. Our results imply an area law $S\left( \mathcal {X(}\Omega \mathcal {)}\right) \lesssim \left| \partial {\Omega }\right|$ when ${\mathcal {X}}$ is the *infinite Ginibre ensemble *with kernel given as

$$\begin{aligned} K_{0}(z,w)=e^{-\frac{\pi }{2}(\left| z\right| ^{2}+\left| w\right| ^{2})}e^{\pi \overline{z}w} \end{aligned}$$

and when ${\mathcal {X}}$ is one of the Ginibre-type ensembles [25], defined with the reproducing kernel of the *n* eigenspace of the Landau operator $L_{z}:=-\partial _{z}\partial _{\overline{z}}+\pi \overline{z} \partial _{\overline{z}}$,

$$\begin{aligned} K_{n}(z,w)=e^{-\frac{\pi }{2}(\left| z\right| ^{2}+\left| w\right| ^{2})}L_{n}(\pi \left| z-w\right| ^{2})e^{\pi \overline{ z}w}\text {.} \end{aligned}$$

Our main result will be stated for *The Weyl–Heisenberg ensemble* ${\mathcal {X}}_{g}$ on ${\mathbb {R}}^{2d}$ introduced in[3] and studied further in [5, 21], a family of DPPs depending on a window function $g\in L^{2}({\mathbb {R}}^{d})$, with correlation kernel

$$\begin{aligned} K_{g}(z,w)={K}_{g}((x,\xi ),(x^{\prime },\xi ^{\prime }))=\int _{{\mathbb {R}} ^{d}}e^{2\pi i(\xi ^{\prime }-\xi )t}g(t-x^{\prime })\overline{g(t-x)}dt \text {.} \end{aligned}$$

When *g* is a Gaussian, $$K_{g}(z,w)$$ becomes a weighted version of $$ K_{0}(z,w)$$ and when *g* is a Hermite function, it becomes a weighted version of $$K_{n}(z,w)$$. More details about these specializations will be given in section 3.

A DPP ${\mathcal {X}}$ is said to be *hyperuniform of Class I* [27, 26, (97) and Table 1], if $\ {\mathbb {V}}\left( {\mathcal {X}}(\Omega \mathcal {)}\right) \lesssim \left| \partial {\Omega } \right|$ or, asymptotically, for a dilated region $R{\Omega }$, if $\ {\mathbb {V}}\left( {\mathcal {X}}(\Omega \mathcal {)}\right) \sim R^{d-1}$ as $R\rightarrow \infty$. As a result of (1.1), area laws for the DPPs considered in this paper will follow as a consequence of their Class I hyperuniformity of rate 1. Hyperuniform states of matter are correlated systems characterized by the suppression of density fluctuations at large scales [3, 14, 15, 26–28]. While the relation (1.1) suggests what seems to be a hitherto unnoticed relation between the concepts of entanglement entropy and of hyperuniformity, similarities between the entanglement entropy and variance fluctuations have been empirically observed in several contexts [11], suggesting that both concepts may be used to quantify the level of supression of fluctuations at large scales typical of a number of physical and mathematical systems known as hyperuniform [26, (97) and Table 1]. The inequality (1.1) is a first step towards a mathematical proof of this hypothesis.

The presentation of this note is organized as follows. The next section contains the concepts of entanglement entropy and number variance for DPPs and proves the inequality (1.1) under the assumptions on $\textrm{trace }\left( T_{\Omega }^{p}\left( 1-T_{\Omega }\right) ^{p}\right)$. The third section introduces some notions about the Weyl–Heisenberg ensemble, and (1.1) is assured to hold for this case, thanks to the bounds of $\textrm{ trace}\left( T_{\Omega }^{p}\left( 1-T_{\Omega }\right) ^{p}\right)$, recently obtained by Marceca and Romero [23]. We then state and prove the bound $S\left( {\mathcal {X}}_{g}\mathcal {(}\Omega \mathcal {)}\right) \lesssim \left| \partial {\Omega }\right|$ on the entanglement entropy of Weyl–Heisenberg ensembles. A lower bound $\left| \partial { \Omega }\right| \lesssim S\left( {\mathcal {X}}_{g}\mathcal {(}\Omega \mathcal {)}\right)$ is also observed to hold under some extra assumptions, and the important examples of Ginibre and of Shirai’s Ginibre-type ensembles on higher Landau levels [25] are used to illustrate the scope of the result on Weyl–Heisenberg ensembles. In the last section, bounds on the entropy using the construction of finite Weyl–Heisenberg ensembles [5] are obtained.

## Entanglement entropy and variance of DPPs

We refer to [20, 21] for precise definitions and background on DPPs. A locally integrable kernel *K*(*z*, *w*) defines the correlation kernel of a determinantal point process (DPP) distributing ${\mathcal {X}}\left( \Omega \right)$ points in $\Omega \subset {\mathbb {R}}^{2d}$, whose *k*-point intensities are given by $\rho _{k}(z_{1},...,z_{k})=\det \left( K(z_{i},z_{j})\right) _{1\le i,j\le k}$. The 1-point intensity of ${\mathcal {X}}$ is then given by $\rho _{1}(z)=K(z,z)$, allowing to compute the expected number of points that fall in $\Omega$ as

$$\begin{aligned} {\mathbb {E}}\left[ \mathcal {X(}\Omega \mathcal {)}\right] {=} \int _{\Omega }K(z,z)dz\text {,} \end{aligned}$$

while the number variance in $\Omega$ is given as (see [13, pg.40] for a detailed proof):

2.1 $$\begin{aligned} {\mathbb {V}}\left( \mathcal {X(}\Omega \mathcal {)}\right) {=}{\mathbb {E}}\left[ \mathcal {X(}\Omega \mathcal {)}^{2}\right] {-}{\mathbb {E}}\left[ {\mathcal {X}}_{g} \mathcal {(}\Omega \mathcal {)}\right] ^{2}{=}\int _{\Omega }K\left( z,z\right) dz{-}\int _{\Omega ^{2}}\left| K\left( z,w\right) \right| ^{2}dzdw\text {.} \nonumber \\ \end{aligned}$$

Consider a compact set $\Omega \subset {\mathbb {R}}^{2d}$. The entanglement entropy $S({\mathcal {X}}(\Omega ))$ measures the degree of entanglement of the DPP ${\mathcal {X}}$ reduced to the region $\Omega$. A DPP satisfies *an area law* if the leading term of the entanglement entropy grows at most proportionally with the measure of the boundary of the partition defining the reduced state [7, 10]. In ${\mathbb {R}}^{2d}=\Omega \cup \Omega ^{c}$ this corresponds to a growth of the order of the perimeter $\left| \delta \Omega \right|$. The set $\Omega \subseteq {{\mathbb {R}}^{2d}}$ is said to have *finite perimeter* if its characteristic function $1_{\Omega }$ is of bounded variation (the concept of ‘area law’ for the entanglement entropy would be, with this terminology, more precisely named as ‘perimeter law’, but we keep up with the traditional terminology). In this case, its perimeter is $\left| \partial \Omega \right| := Var (1_{\Omega })$.

Our analysis is based on associating to the kernel of ${\mathcal {X}}$, *K*(*z*, *w*) (a locally integrable reproducing kernel of a Hilbert space $H\subset L^{2}\left( {\mathbb {R}}^{2d}\right)$), the following operator:

$$\begin{aligned} (T_{\Omega }f)(z)=\int _{\Omega }f(w)\overline{K(z,w)}dw\text {,} \end{aligned}$$

where *dw* stands for Lebesgue measure, mapping *f* to a smooth function in $L^{2}\left( {\mathbb {R}}^{2d}\right)$ with most of its energy concentrated in the region $\Omega$. Since $$\Omega \subset {\mathbb {R}}^{2d}\ $$is compact and *K*(*z*, *w*) locally integrable, $T_{\Omega }$ is a compact positive (self-adjoint) operator of trace class, and one can invoke the spectral theorem to assure that $T_{\Omega }$ is diagonalized by an orthonormal set of eigenfunctions $\{e_{n}^{\Omega }(z):n\ge 1\}$ with corresponding eigenvalues $\{\lambda _{n}^{\Omega }:n\ge 1\}$ ordered non-increasingly. The operator is positive and bounded by 1 (see [2, Lemma 2.1] for details in the Weyl–Heisenberg case).

For the definition of entanglement entropy of a DPP on a region $\Omega$ we will use the result in Proposition 7.2 of [7].

### Definition 2.1

The entanglement entropy $S({\mathcal {X}}(\Omega ))$ of the DPP ${\mathcal {X}}$ on a compact set $\Omega \subset {\mathbb {R}}^{2d}$ is defined in terms of $T_{\Omega }$ as

$$\begin{aligned} S({\mathcal {X}}(\Omega ))=\textrm{trace}(f(T_{\Omega }))\text {,} \end{aligned}$$

where

2.2 $$\begin{aligned} f(x)=-x\ln x-(1-x)\ln (1-x)\text {.} \end{aligned}$$

The traces of $T_{\Omega }$ and $T_{\Omega }^{2}$ are given by ($K\left( z,z\right) =1$)

2.3 $$\begin{aligned}&\textrm{trace}(T_{\Omega })=\int _{\Omega }K(z,z)dz={\mathbb {E}}\left[ \mathcal {X(}\Omega \mathcal {)}\right] ={|\Omega |}=\sum _{n\ge 1}{\lambda _{n}^{\Omega }}, \end{aligned}$$

2.4 $$\begin{aligned}&\textrm{trace}(T_{\Omega }^{2})=\int _{\Omega ^{2}}\left| K\left( z,w\right) \right| ^{2}dzdw=\sum _{n\ge 1}{(\lambda _{n}^{\Omega }})^{2} \text {.} \end{aligned}$$

and the number variance of ${\mathcal {X}}(\Omega )$, according to (2.1), by

2.5 $$\begin{aligned} {\mathbb {V}}\left( {\mathcal {X}}(\Omega )\right) =\textrm{trace}(T_{\Omega })- \textrm{trace}(T_{\Omega }^{2})=\sum _{n\ge 1}{\lambda _{n}^{\Omega }-} \sum _{n\ge 1}{(\lambda _{n}^{\Omega }})^{2}\text {.} \end{aligned}$$

It has been drawn to the attention of the author by Gröchenig [17] that, for $x\in \left[ 0,1\right]$, the following inequality can be easily proved:

2.6 $$\begin{aligned} 4x(x-1)\le \frac{1}{\log 2}f(x)\text {.} \end{aligned}$$

where $f(x)=-x\ln x-(1-x)\ln (1-x)$, so that ${\mathbb {V}}\left( {\mathcal {X}} (\Omega )\right) =\textrm{trace}\left( T_{\Omega }-T_{\Omega }^{2}\right) \le \frac{1}{4\log 2}\textrm{trace}(f(T_{\Omega }))$. This leads to a lower bound for the entanglement entropy

2.7 $$\begin{aligned} {\mathbb {V}}\left( {\mathcal {X}}(\Omega )\right) \lesssim S\left( \mathcal {X(} \Omega \mathcal {)}\right) \text {.} \end{aligned}$$

Inequality (2.7) has been used before to show the violation of the area law by fermionic process (see [16] and the references therein, where also upper inequalities for the entropy in terms of the variance, with a log correction term, are obtained). For $x\in \left[ 0,1 \right]$ one cannot expect a pointwise upper bound for $f(x)=-x\ln x-(1-x)\ln (1-x)$ as a constant times $x(x-1)$, due to the singularities of *f*(*x*). Nevertheless, under a boundedness conditions on the so-called Schatten *p*-norms of $T_{\Omega }-T_{\Omega }^{2}$, it is possible to prove an upper bound by relating the trace of the functions of positive self-adjoint operators bounded by 1.

Our main results will depend on the following inequality, conditioned to a bound on the Schatten *p*-norms of $T_{\Omega }-T_{\Omega }^{2}$.

### Proposition 2.2

Let ${\mathcal {X}}$ be a DPP on ${\mathbb {R}}^{2d}$ such that the associated operator $T_{\Omega }$ is self-adjoint, positive, bounded by 1 and is of trace class satisfying, for $0<p<1$,

2.8 $$\begin{aligned} \textrm{trace}\left( T_{\Omega }^{p}\left( 1-T_{\Omega }\right) ^{p}\right) \le C\text {,} \end{aligned}$$

where *C* depends on $\Omega$ and *p*. Then the entanglement entropy and the variance of $\mathcal {X(}\Omega \mathcal {)}$ satisfy

2.9 $$\begin{aligned} {\mathbb {V}}\left( {\mathcal {X}}(\Omega )\right) \lesssim S\left( \mathcal {X(} \Omega \mathcal {)}\right) \lesssim {\mathbb {V}}\left( {\mathcal {X}}(\Omega )\right) \text {.} \end{aligned}$$

### Proof

Observe that $f(x)=-x\ln x-(1-x)\ln (1-x)$ belongs to the class of continuous function such that $$\left| f(t)\right| =O(t^{p})\ $$and $\left| f(1-t)\right| =O(t^{p})$ as $t\rightarrow 0$ with $p>0$. Strongly inspired by the idea of [7, Theorem 6.2], we will prove that, for *f* in this class, if $\Omega \subset {\mathbb {R}}^{2d}$ is compact, then

$$\begin{aligned} \textrm{trace}(f(T_{\Omega }))\lesssim {\mathbb {V}}\left( {\mathcal {X}}(\Omega )\right) \text {.} \end{aligned}$$

The proof will use that $\textrm{trace}$ is a positive linear functional, in the sense that if $f\le g$ then $\textrm{trace}\left( f\right) \le \textrm{ trace}\left( g\right)$, and relate $\textrm{trace}(f(T_{\Omega }))$ to ${\mathbb {V}}\left( {\mathcal {X}}(\Omega )\right)$ using the identity (2.5), first for polynomials vanishing at 0 and 1 and then for functions of the form $f(z)=g(x)h_{p}(x)$ with $$h_{p}(x)=x^{p}(1-x)^{p}\ $$and $g\in C(\left[ 0,1\right] )$ such that $g(0)=g(1)=0$, using polynomial approximation.

**Step 1.** In this step we prove that $\textrm{trace}(P_{n}(T_{\Omega }))\lesssim {\mathbb {V}}\left( {\mathcal {X}}(\Omega )\right)$, where $P_{n}$ is a polynomial of degree *n*, such that $P(0)=P(1)=0$. For $k\ge 1$,

$$\begin{aligned} \textrm{trace}\left( T_{\Omega }^{k}\right) -\textrm{trace}\left( T_{\Omega }^{k+1}\right) =\textrm{trace}\left( T_{\Omega }^{k-1}\left( T_{\Omega }-T_{\Omega }^{2}\right) \right) \text {.} \end{aligned}$$

Since $T_{\Omega }-T_{\Omega }^{2}$ is a non-negative defined operator, we can use the inequality $\textrm{trace}\left( AB\right) \le \left\| A\right\| \textrm{trace}$(*B*), together with $\left\| T_{\Omega }^{k-1}\right\| \le 1$, to obtain

$$\begin{aligned} \textrm{trace}\left( T_{\Omega }^{k}\right) -\textrm{trace}\left( T_{\Omega }^{k+1}\right) \le \textrm{trace}\left( T_{\Omega }-T_{\Omega }^{2}\right) = {\mathbb {V}}\left( {\mathcal {X}}(\Omega )\right) \text {.} \end{aligned}$$

Since a general polynomial vanishing at 0 and 1 can be written as linear combinations of $x^{k}-x^{k+1}$, we write

$$\begin{aligned} P_{n}(x)=\sum _{k=0}^{n}a_{k}\left( x^{k}-x^{k+1}\right) \end{aligned}$$

and the above gives, by linearity,

$$\begin{aligned} \textrm{trace}\left( P_{n}(T_{\Omega })\right) =\sum _{k=0}^{n}a_{k}\left( \textrm{trace}\left( T_{\Omega }^{k}\right) -\textrm{trace}\left( T_{\Omega }^{k+1}\right) \right) \lesssim {\mathbb {V}}\left( {\mathcal {X}}(\Omega )\right) \text {.} \end{aligned}$$

**Step 2. **We show that, for every $$p>0$$, $$\textrm{trace}\left( h_{p}\left( T_{\Omega }\right) \right) $$ is bounded, where $$ h_{p}(x)=x^{p}(1-x)^{p}$$, $$0<x<1$$. For $$p\ge 1$$ and $$0<x<1$$, we have $$ x^{p}(1-x)^{p}\le x(1-x)$$ and

$$\begin{aligned} \textrm{trace}\left( h_{p}\left( T_{\Omega }\right) \right) =\textrm{trace} \left( T_{\Omega }^{p}\left( 1-T_{\Omega }\right) ^{p}\right) \le \textrm{ trace}\left( T_{\Omega }-T_{\Omega }^{2}\right) ={\mathbb {V}}\left( {\mathcal {X}} (\Omega )\right) \text {.} \end{aligned}$$

For $0<p<1$ and $0<x<1$, it follows from the hypothesis (2.8) that $\textrm{trace}\left( h_{p}\left( T_{\Omega }\right) \right)$ is bounded by $C>0$. We have thus

$$\begin{aligned} \textrm{trace}\left( h_{p}\left( T_{\Omega }\right) \right) \le C_{0}\text {, } \end{aligned}$$

where $$C_{0}=\max \{{\mathbb {V}}\left( {\mathcal {X}}(\Omega )\right) ,C\}$$.

**Step 3. **For the extension to continuous functions *f* such that $$ \left| f(t)\right| =O(t^{p})\ $$and $\left| f(1-t)\right| =O(t^{p})$ as $t\rightarrow 0$ with $p>0$, we use a polynomial approximation argument as in [7, Theorem 6.2]. For a $p>0$ one can write *f* as $f(z)=g(x)h_{p}(x)$ with $$h_{p}(x)=x^{p}(1-x)^{p}\ $$and $g\in C( \left[ 0,1\right] )$ such that $g(0)=g(1)=0$. Given $\epsilon >0$ we can invoke the Weierstrass approximation theorem to find a polynomial *P*(*x*) such that $P(0)=P(1)=0$ and $\left| g-P\right| <\epsilon$. Thus, $\textrm{trace}\left( f\left( T_{\Omega }\right) \right) =\textrm{trace} \left( gh_{p}\left( T_{\Omega }\right) \right)$ and the polynomial approximation of *g* by *P* allows one to write $g\le P+\epsilon$ and

2.10 $$\begin{aligned} \textrm{trace}\left( f\left( T_{\Omega }\right) \right) {=}\textrm{trace} \left( gh_{p}\left( T_{\Omega }\right) \right) {\le } \textrm{trace}\left( Ph_{p}\left( T_{\Omega }\right) \right) {+}\epsilon \textrm{trace}\left( h_{p}\left( T_{\Omega }\right) \right) \text {.} \end{aligned}$$

Combining with Step 2, we arrive at

2.11 $$\begin{aligned} \textrm{trace}\left( f\left( T_{\Omega }\right) \right) \le \textrm{trace} \left( Ph_{p}\left( T_{\Omega }\right) \right) +\epsilon C_{0}\text {.} \end{aligned}$$

Since $$P(0)=P(1)=0$$, there exists a polynomial $$P_{1}(x)$$ such that $$ P(x)=P_{1}(x)h_{1}(x)$$, leading to $$P(x)h_{p}(x)=P_{1}(x)h_{p}(x)h_{1}(x)$$. This allows to control $$\textrm{trace}\left( Ph_{p}\left( T_{\Omega }\right) \right) $$, by writing $$g(x)=P_{1}(x)h_{p}(x)$$ and invoking Weierstrass approximation of $$g(x)\ $$by another polynomial $$P_{2}(x)$$. For an $$\epsilon _{1}>0$$ we obtain, since $$g\le P_{2}+\epsilon _{1}$$,

2.12 $$\begin{aligned} \textrm{trace}\left( Ph_{p}\left( T_{\Omega }\right) \right) =\textrm{trace} \left( gh_{1}\left( T_{\Omega }\right) \right) \le \textrm{trace}\left( P_{2}h_{1}\left( T_{\Omega }\right) \right) +\epsilon _{1}\textrm{trace} \left( h_{1}\left( T_{\Omega }\right) \right) \text {.} \nonumber \\ \end{aligned}$$

By Step 1, since $P_{2}(x)h_{1}(x)$ is a polynomial,

$$\begin{aligned} \textrm{trace}\left( P_{2}h_{1}\left( T_{\Omega }\right) \right) \lesssim {\mathbb {V}}\left( {\mathcal {X}}(\Omega )\right) \text {.} \end{aligned}$$

Observing that

$$\begin{aligned} \textrm{trace}\left( h_{1}\left( T_{\Omega }\right) \right) =\textrm{trace} \left( T_{\Omega }-T_{\Omega }^{2}\right) ={\mathbb {V}}\left( {\mathcal {X}} (\Omega )\right) \text {,} \end{aligned}$$

then (2.12) leads to

$$\begin{aligned} \textrm{trace}\left( Ph_{p}\left( T_{\Omega }\right) \right) \lesssim {\mathbb {V}}\left( {\mathcal {X}}(\Omega )\right) +\epsilon _{1}{\mathbb {V}}\left( {\mathcal {X}}(\Omega )\right) \text {.} \end{aligned}$$

It follows from (2.11) that

$$\begin{aligned} \textrm{trace}\left( f\left( T_{\Omega }\right) \right) \lesssim {\mathbb {V}} \left( {\mathcal {X}}(\Omega )\right) +\epsilon _{1}{\mathbb {V}}\left( {\mathcal {X}} (\Omega )\right) +\epsilon C_{0}\text {.} \end{aligned}$$

Since $\epsilon$ and $\epsilon _{1}$ are at our disposal, this implies $\textrm{trace}\left( f\left( T_{\Omega }\right) \right) \lesssim {\mathbb {V}} \left( {\mathcal {X}}(\Omega )\right)$. $\square$

## Entanglement entropy of Weyl–Heisenberg ensembles

The main result will be stated in terms of *Weyl–Heisenberg ensembles*. This includes as special cases the Ginibre ensemble and its higher Landau levels versions. To motivate the choice of the correlation kernel, recall that for $z=(x,\xi )\in {\mathbb {R}}^{2d}$, the short-time Fourier transform of a function *f* with respect to a window function $g\in L^{2}({\mathbb {R}} ^{d})$ is defined as [18]:

3.1 $$\begin{aligned} \mathcal {V}_{g}f(x,\xi )=\int _{{\mathbb {R}}^{d}}f(t)\overline{g(t-x)}e^{-2\pi i\xi t}dt\text {.} \end{aligned}$$

For $d=1$ and $g(t)=h_{0}(t)=2^{1/4}e^{-\pi t^{2}}$, then, writing $z=x+i\xi$, then $$\mathcal {V}_{h_{0}}f(x,-\xi )=e^{-i\pi x\xi }e^{-\frac{\pi }{2} \left| z\right| ^{2}}Bf(z)\ $$where *Bf*(*z*) is the Bargmann-Fock transform

$$\begin{aligned} Bf(z)=2^{\frac{1}{4}}\int _{{\mathbb {R}}}f(t)e^{2\pi tz-\pi t^{2}-\frac{\pi }{2} z^{2}}dt\text {,} \end{aligned}$$

which maps $L^{2}({\mathbb {R}})$ onto the Fock space of entire functions, whose reproducing kernel is the kernel of the infinite Ginibre ensemble and which, as a ressult, can be seen as a weighted version of $\mathcal {V} _{h_{0}}\left( L^{2}({\mathbb {R}})\right)$. For choices of *g* within the family of Hermite functions $h_{n}(t)$, defined as in (3.5), one obtains a sequence of transforms defined by $\mathcal {V}_{h_{n}}f(x,-\xi )=e^{-i\pi x\xi }e^{-\frac{\pi }{2}\left| z\right| ^{2}}B^{(n)}(z)$, and mapping $L^{2}({\mathbb {R}})$ onto the eigenspaces of the Landau levels operator, which are weighted versions of $\mathcal {V}_{h_{n}}\left( L^{2}( {\mathbb {R}})\right)$ [1, 3, 5].

*The Weyl–Heisenberg ensemble*, introduced in[3] and studied further in [5, 21], is the family of DPPs $\mathcal {X }_{g}$ on ${\mathbb {R}}^{2d}$, with correlation kernel equal to the reproducing kernel of $\mathcal {V}_{g}L^{2}({\mathbb {R}}^{d})$:

3.2 $$\begin{aligned} K_{g}(z,w)={K}_{g}((x,\xi ),(x^{\prime },\xi ^{\prime }))=\int _{{\mathbb {R}} ^{d}}e^{2\pi i(\xi ^{\prime }-\xi )t}g(t-x^{\prime })\overline{g(t-x)}dt \text {,} \end{aligned}$$

for some non-zero function $g\in L^{2}({\mathbb {R}}^{d})$ with $$\left\| g\right\| _{L^{2}({\mathbb {R}}^{d})}=1\ $$and $(x,\xi ),(x^{\prime },\xi ^{\prime })\in {\mathbb {R}}^{2d}$. For *g* a Hermite function, Weyl–Heisenberg ensembles lead to the Ginibre type ensembles for higher Landau levels [3, 25] (see the remark below) and to the Heisenberg family of DPPs [24]. The complex Ginibre ensemble as the prototypical Weyl–Heisenberg ensemble follows by setting $d=1$ and choosing *g* in  (3.2) to be the Gaussian $h_{0}(t)=2^{1/4}e^{-\pi t^{2}}$. The resulting kernel is

$$\begin{aligned} {K}_{h_{0}}(z,w)=e^{i\pi (x^{\prime }\xi ^{\prime }-x\xi )}e^{-\frac{\pi }{2} (\left| z\right| ^{2}+\left| w\right| ^{2})}e^{\pi \overline{ z}w},\qquad z=x+i\xi ,\,w=x^{\prime }+i\xi ^{\prime }. \end{aligned}$$

Modulo a phase factor, this is the kernel of the *infinite Ginibre ensemble* $K_{0}(z,w)=e^{-\frac{\pi }{2}(\left| z\right| ^{2}+\left| w\right| ^{2})}e^{\pi \overline{z}w}$. Choosing $h_{n}(t)$ a Hermite function, a similar relation holds between ${K} _{h_{n}}(z,w)$ and $K_{n}(z,w)$.

The area law is obtained for Berezin-Toeplitz operators on compact Kaehler manifolds and for the Bargmann transform (including thus the first Landau level case of the Ginibre DPP) in [7], but the relation with the variance is not made explicit. In [22], a proportionality relation between the entanglement entropy and the number variance has been obtained for the *finite* Ginibre ensemble (it is unclear at the moment if the methods in this note can handle finite DPPs since, in such cases, the higher order traces may be difficult to control). For a discussion of the relations between entanglement entropy and variance fluctuations in a broad sense, see [11].

### Theorem 3.1

Let $$\Omega \subset {\mathbb {R}}^{2d}\ $$compact.Let $K_{g}(z,w)$ be the kernel of a Weyl–Heisenberg ensemble ${\mathcal {X}}_{g}$ with *g* satisfying, for some $s\ge 1/2$,

3.3 $$\begin{aligned} C_{g}=\left[ \int _{{\mathbb {R}}^{2d}}\left| V_{g}g(z)\right| dz\right] ^{2}\int _{{\mathbb {R}}^{2d}}(1+|z|)^{2s}\left| V_{g}g(z)\right| ^{2}dz<\infty \text {.} \end{aligned}$$

Then the entanglement entropy of the Weyl–Heisenberg ensemble on $\Omega$ satisfies the area law

$$\begin{aligned} S\left( {\mathcal {X}}_{g}\mathcal {(}\Omega \mathcal {)}\right) \lesssim \left| \partial {\Omega }\right| \text {.} \end{aligned}$$

### Proof

We follow [23, (2.6)] and consider the Schatten quasinorm of the Hankel operator such that $H^{*}H=T_{\Omega }-T_{\Omega }^{2}$. Then

$$\begin{aligned} \left\| H\right\| _{\widetilde{p}}^{\widetilde{p}}=\textrm{trace} \left( \left( H^{*}H\right) ^{\frac{1}{2}}\right) ^{\widetilde{p}}= \textrm{trace}\left( T_{\Omega }^{\widetilde{p}}\left( 1-T_{\Omega }\right) ^{\widetilde{p}}\right) ^{\frac{1}{2}}=\textrm{trace}\left( h_{\widetilde{p} /2}\left( T_{\Omega }\right) \right) \text {,} \end{aligned}$$

where $h_{p}(x)=x^{p}(1-x)^{p}$. Thus, the results for $\widetilde{p}<2$ in Proposition 3.1 in [23], assuming (3.3), assure, writing $p=\frac{1 }{2}\widetilde{p}$ that, for $0<p<1$, $\textrm{trace}\left( h_{\widetilde{p} /2}\left( T_{\Omega }\right) \right) =\textrm{trace}\left( h_{p}\left( T_{\Omega }\right) \right) =\textrm{trace}\left( T_{\Omega }^{p}\left( 1-T_{\Omega }\right) ^{p}\right)$ is bounded by $C\left| \partial { \Omega }\right| >0$. Thus, we can apply Proposition 2.2 to yield

$$\begin{aligned} S\left( {\mathcal {X}}_{g}\mathcal {(}\Omega \mathcal {)}\right) =\textrm{trace} (f(T_{\Omega }))\lesssim {\mathbb {V}}\left( {\mathcal {X}}_{g}(\Omega )\right) = \textrm{trace}\left( T_{\Omega }-T_{\Omega }^{2}\right) \text {.} \end{aligned}$$

Let $\varphi \in {L^{1}({{\mathbb {R}}^{d}})}$ with $\int \varphi =1$. Then, for a set $\Omega$ of finite perimeter $\left| \partial {\Omega } \right|$, Lemma 3.2 in [2] gives

$$\begin{aligned} \Vert 1_{\Omega }*\varphi -1_{\Omega }\Vert _{L^{1}({{\mathbb {R}}^{2d}} )}\le \left| \partial {\Omega }\right| \int _{{\mathbb {R}} ^{2d}}\left| z\right| \left| \varphi (z)\right| dz\text {.} \end{aligned}$$

Applying this inequality with $\varphi (z)=\left| V_{g}g(z)\right| ^{2}$ and observing that $K_{g}(z,w)=V_{g}g(z-w)$ leads to

$$\begin{aligned} \textrm{trace}\left( T_{\Omega }-T_{\Omega }^{2}\right)= & {} \left| \int _{\Omega }\int _{\Omega }\varphi (z-w)dzdw-\int _{\Omega }dz\right| \\= & {} \left| \int _{\Omega }\left( 1_{\Omega }*\varphi (w)-1_{\Omega }\right) dw\right| \le \Vert 1_{\Omega }*\varphi -1_{\Omega }\Vert _{L^{1}({{\mathbb {R}}^{2d}})}\le C\left| \partial {\Omega } \right| \text {,} \end{aligned}$$

where $C=\int _{{\mathbb {R}}^{2d}}\left| z\right| \left| V_{g}g(z)\right| ^{2}dz$. This last bound has been obtained in a different form in [8]. The more direct proof presented is implicit in [2].$\square$

### Example 3.2

The Landau operator acting on the Hilbert space $L^{2}\left( {\mathbb {C}},e^{- \frac{\pi }{2}\left| z\right| ^{2})}\right)$ can be defined as

3.4 $$\begin{aligned} L_{z}:=-\partial _{z}\partial _{\overline{z}}+\pi \overline{z}\partial _{ \overline{z}}\text {.} \end{aligned}$$

The spectrum of $L_{z}$ is given by $\sigma (L_{z})=\{\pi n:n=0,1,2,\ldots \}$. The eigenspaces have associated reproducing kernel [6]

$$\begin{aligned} K_{n}(z,w)=L_{n}(\pi \left| z-w\right| ^{2})e^{\pi \overline{z}w} \text {,} \end{aligned}$$

where $$L_{n}\ $$is a Laguerre polynomial. Let the window *g* of the Weyl–Heisenberg kernel be a Hermite function

3.5 $$\begin{aligned} h_{n}(t)=\frac{2^{1/4}}{\sqrt{n!}}\left( \frac{-1}{2\sqrt{\pi }}\right) ^{n}e^{\pi t^{2}}\frac{d^{n}}{dt^{n}}\left( e^{-2\pi t^{2}}\right) ,\qquad n\ge 0. \end{aligned}$$

Then

$$\begin{aligned} {K}_{h_{n}}(z,w)= & {} e^{i\pi (x^{\prime }\xi ^{\prime }-x\xi )}e^{-\frac{\pi }{2} (\left| z\right| ^{2}+\left| w\right| ^{2})}L_{n}(\pi \left| z-w\right| ^{2})e^{\pi \overline{z}w},\\ {}{} & {} \qquad z=x+i\xi ,\,w=x^{\prime }+i\xi ^{\prime }\text {.} \end{aligned}$$

Now, from Theorem 2.2, denoting by ${\mathcal {X}}_{n}$ the DPP associated to the *nth* Landau level,

$$\begin{aligned} S({\mathcal {X}}_{n}(\Omega ))\lesssim \left| \partial {\Omega }\right| \text {.} \end{aligned}$$

Moreover, from [25, Theorem 1.1] (see also [9, p. 3] for an alternative proof), one has $S({\mathcal {X}} _{n}(D_{r}))\sim C_{n}r$ as $r\rightarrow \infty$. It follows that

$$\begin{aligned} S({\mathcal {X}}_{n}(D_{r}))\sim Cr\text {,} \end{aligned}$$

as $r\rightarrow \infty$, for some constant *C*. This is an area law (in ${\mathbb {R}}^{2}$) for the entanglement entropy of integer quantum Hall states modelled by DPP on higher Landau levels (see also the limit case $\beta =1$ in Theorem 2.5 of [12]).

### Remark 3.3

Now, putting together Proposition 4.2 and Lemma 4.3 of [8], and (2.7) we realize, that, under certain conditions on *g*, we have $\left| \partial {\Omega }\right| \lesssim {\mathbb {V}}\left( \mathcal {X }_{g}(\Omega )\right)$. Thus, under the conditions of Proposition 4.2 and Lemma 4.3 in [8], we have a double bound for the growth of the entanglement entropy of the Weyl–Heisenberg ensemble on $\Omega$:

$$\begin{aligned} \left| \partial {\Omega }\right| \lesssim S\left( {\mathcal {X}}_{g} \mathcal {(}\Omega \mathcal {)}\right) \lesssim \left| \partial {\Omega } \right| \text {.} \end{aligned}$$

The condition (2.8) has been verified in [7] under the assumption of Gaussian decay of the kernel, and the analysis includes fermionic states on a Kähler manifold and the infinite Ginibre ensemble. For the kernel corresponding to the Weyl–Heisenberg ensemble, the first bounds were obtained in [8] and the moderate decay (3.3 ) required considerable technical work [23].

### Remark 3.4

For general *d*, class II hyperuniformity is characterized by the following asymptotic growth of the variance on a compact region $\Omega \subset {\mathbb {R}}^{d}$ dilated by $L>0$

$$\begin{aligned} {\mathbb {V}}\left( {\mathcal {X}}(L\Omega )\right) \sim C_{\Omega }L^{d-1}\log L , \quad L\rightarrow \infty \text {.} \end{aligned}$$

Thus, just using inequality (2.7), (which holds without assumptions on the kernel, since it follows from an inequality valid pointwise), we conclude at once the following: *for a DPP X in the Class II hyperuniformity*,

$$\begin{aligned} S\left( {\mathcal {X}}(L\Omega )\right) \ge O(L^{d-1}\log L)\text {,} \end{aligned}$$

as $L\rightarrow \infty$ leading to the *violation of the area law*, due to the $\log L$ correction. Thus, every *Class II hyperuniform DPP violates the area law.*

## Entanglement entropy and finite Weyl–Heisenberg ensembles

A feature of the Weyl–Heisenberg ensemble is the possibility of constructing finite-dimensional DPPs with first point intensity converging to the indicator domain of a pre-defined compact region $$\Omega $$. Details of such finite dimensional constructions are given in [5], where it is shown, in the Hermite window case, that the resulting processes are closely related to the finite polyanalytic Ginibre ensembles of [19]. We will now sketch the construction of finite Weyl–Heisenberg ensembles. Since $$ \{e_{n}^{\Omega }(z):n\ge 1\}$$ spans the space with reproducing kernel $$ K_{g}(z,w)$$, we have

$$\begin{aligned} K_{g}(z,w)=\sum _{n\ge 1}e_{n}^{\Omega }(z)\overline{e_{n}^{\Omega }(w)} \text {.} \end{aligned}$$

Now we define the finite Weyl–Heisenberg ensemble as follows (see the introduction of [5] for details).

### Definition 4.1

Let $$N_{\Omega }=\left\lfloor \Omega \right\rfloor \ $$be the smallest integer greater than or equal to $\left| \Omega \right|$. * The finite Weyl–Heisenberg ensemble *${\mathcal {X}}_{g}^{N_{\Omega }}$ is the determinantal point process (DPP) associated with the truncated kernel

$$\begin{aligned} K_{g}^{N_{\Omega }}(z,w)=\sum _{n=1}^{N_{\Omega }}e_{n}^{\Omega }(z)\overline{ e_{n}^{\Omega }(w)}\text {.} \end{aligned}$$

### Example 4.2

Consider the Gaussian $h_{0}(t)=2^{1/4}e^{-\pi t^{2}}$ leading to the infinite Ginibre ensemble kernel

4.1 $$\begin{aligned} K_{h_{0}}(z,w)=e^{i\pi (x^{\prime }\xi ^{\prime }-x\xi )}e^{-\frac{\pi }{2} (\left| z\right| ^{2}+\left| w\right| ^{2})}e^{\pi z \overline{w}}\text {.} \end{aligned}$$

Denote by $\left| D_{R}\right| =\pi R^{2}$ the area of the disc. The eigenfunctions of

$$\begin{aligned} (T_{D_{R}}f)(z)=\int _{D_{R}}f(w)\overline{K_{h_{0}}(z,w)}dw\text {,} \end{aligned}$$

are $e_{n+1}^{N_{D_{R}}}(z)=\left( \frac{\pi ^{j}}{j!}\right) ^{\frac{1}{2} }e^{-i\pi x\xi }e^{-\frac{\pi }{2}\left| z\right| ^{2}}z^{n}$. The corresponding kernel of the finite Weyl–Heisenberg ensemble on $D_{R}$ is then

4.2 $$\begin{aligned} K_{h_{0}}^{N_{D_{R}}}(z,w)=e^{i\pi (x^{\prime }\xi ^{\prime }-x\xi )}e^{- \frac{\pi }{2}(\left| z\right| ^{2}+\left| w\right| ^{2})}\sum _{n=0}^{N_{D_{R}}-1}\frac{\left( \pi z\overline{w}\right) ^{n}}{n!} \text {,} \end{aligned}$$

where $N_{D_{R}}=\left\lfloor \pi R^{2}\right\rfloor$. This is, modulo a phase factor, the kernel of the finite Ginibre ensemble, obtained by truncating the expansion of the exponential $e^{\pi z\overline{w}}$.

We now provide a bound on $S({\mathcal {X}}_{g}(\Omega ))$ involving the number of points of ${\mathcal {X}}_{g}^{N_{\Omega }}$ that in average fall in $$\Omega \ $$and which can be explicitly computed in terms of the first eigenvalues of $T_{\Omega }$.

### Theorem 4.3

Let $$\Omega \subset {\mathbb {R}}^{2d}\ $$compact and *g* satisfying (3.3). The entanglement entropy of the Weyl–Heisenberg ensemble on $\Omega$ satisfies

$$\begin{aligned} S\left( {\mathcal {X}}_{g}\mathcal {(}\Omega \mathcal {)}\right) \lesssim N_{\Omega }-{\mathbb {E}}\left( {\mathcal {X}}_{g}^{N_{\Omega }}(\Omega )\right) \end{aligned}$$

or

$$\begin{aligned} S\left( {\mathcal {X}}_{g}\mathcal {(}\Omega \mathcal {)}\right) \lesssim N_{\Omega }{-}\sum _{n=1}^{N_{\Omega }}\lambda _{n}^{\Omega }\text {.} \end{aligned}$$

### Proof

The 1-point intensity of ${\mathcal {X}}_{g}^{N_{\Omega }}$ is

$$\begin{aligned} \rho _{1}^{N_{\Omega }}(z)=K_{g}^{N_{\Omega }}\left( z,z\right) =\sum _{n=1}^{N_{\Omega }}\left| e_{n}^{\Omega }(z)\right| ^{2}\text {. } \end{aligned}$$

Thus,

4.3 $$\begin{aligned} {\mathbb {E}}\left( {\mathcal {X}}_{g}^{N_{\Omega }}(\Omega )\right)= & {} \int _{\Omega }K_{g}^{N_{\Omega }}(z,z)dz \nonumber \\= & {} \sum _{n=1}^{N_{\Omega }}\int _{\Omega }\left| e_{n}^{\Omega }(z)\right| ^{2}\,dz=\sum _{n=1}^{N_{\Omega }}{\lambda _{n}^{\Omega }} \end{aligned}$$

and

4.4 $$\begin{aligned} {\mathbb {V}}\left( {\mathcal {X}}_{g}(\Omega )\right)= & {} \sum _{n\ge 1}{\lambda _{n}^{\Omega }}-\sum _{n\ge 1}{(\lambda _{n}^{\Omega }})^{2}=\sum _{n=1}^{N_{ \Omega }}\lambda _{n}^{\Omega }(1-\lambda _{n}^{\Omega })+\sum _{n>N_{\Omega }}\lambda _{n}^{\Omega }(1-\lambda _{n}^{\Omega }) \nonumber \\\le & {} \sum _{n=1}^{N_{\Omega }}(1-\lambda _{n}^{\Omega })+\sum _{n>N_{\Omega }}\lambda _{n}^{\Omega } \nonumber \\= & {} N_{\Omega }-\sum _{n=1}^{N_{\Omega }}\lambda _{n}^{\Omega }+\textrm{trace} (T_{\Omega })-\sum _{n=1}^{N_{\Omega }}\lambda _{n}^{\Omega } \nonumber \\\le & {} 2N_{\Omega }-2{\mathbb {E}}\left( {\mathcal {X}}_{g}^{N_{\Omega }}(\Omega )\right) \text {.} \end{aligned}$$

The result follows from the upper bound $S\left( {\mathcal {X}}_{g}\mathcal {(} \Omega \mathcal {)}\right) \lesssim {\mathbb {V}}\left( {\mathcal {X}}_{g}(\Omega )\right)$.$\square$

We finally bound the entanglement entropy of the Weyl–Heisenberg ensemble on $\Omega$ by the deviation of the 1-point intensity $\rho _{1}^{N_{\Omega }}(z)$ of the finite Weyl–Heisenberg ensemble ${\mathcal {X}}_{g}^{N_{\Omega }}$ from the flat density $1_{\Omega }$.

### Theorem 4.4

Let $$\Omega \subset {\mathbb {R}}^{2d}\ $$compact and *g* satisfying (3.3). The entanglement entropy of the Weyl–Heisenberg ensemble on $\Omega$ satisfies

$$\begin{aligned} \int _{{\mathbb {R}}^{2d}}\left| \rho _{1}^{N_{\Omega }}(z)-1_{\Omega }(z)\right| \,dz\lesssim S\left( {\mathcal {X}}_{g}\mathcal {(}\Omega \mathcal {)}\right) \lesssim \int _{{\mathbb {R}}^{2d}}\left| \rho _{1}^{N_{\Omega }}(z)-1_{\Omega }(z)\right| \,dz\text {.} \end{aligned}$$

### Proof

We start with inequality (4.4) and then proceed as in the proof of Theorem 1.6 in [4]:

$$\begin{aligned} {\mathbb {V}}\left( \mathcal {X(}\Omega \mathcal {)}\right)\le & {} \sum _{n=1}^{N_{\Omega }}(1-\lambda _{n}^{\Omega })+\sum _{n>N_{\Omega }}\lambda _{n}^{\Omega } \\= & {} \int _{{\mathbb {R}}^{2d}-\Omega }\rho _{1}^{N_{\Omega }}(z)\,dz+\left( \textrm{trace}(T_{\Omega })-\int _{\Omega }\rho _{1}^{N_{\Omega }}(z)\,dz\right) \\= & {} \int _{{\mathbb {R}}^{2d}-\Omega }\left| \rho _{1}^{N_{\Omega }}(z)-1_{\Omega }(z)\right| \,dz+\int _{\Omega }\left| \rho _{1}^{N_{\Omega }}(z)-1_{\Omega }(z)\right| \,dz \\= & {} \int _{{\mathbb {R}}^{2d}}\left| \rho _{1}^{N_{\Omega }}(z)-1_{\Omega }(z)\right| \,dz\text {.} \end{aligned}$$

The result follows from (2.9). The lower bound of the variance follows from a related argument, which is contained in the Steps 2 and 3 of the proof of Theorem 1.5 in [4].$\square$

### Remark 4.5

To obtain the previous theorem, we have proved that

$$\begin{aligned} \int _{{\mathbb {R}}^{2d}}\left| \rho _{1}^{N_{\Omega }}(z)-1_{\Omega }(z)\right| \,dz\lesssim {\mathbb {V}}\left( \mathcal {X(}\Omega \mathcal {)} \right) \lesssim \int _{{\mathbb {R}}^{2d}}\left| \rho _{1}^{N_{\Omega }}(z)-1_{\Omega }(z)\right| \,dz\text {.} \end{aligned}$$

This holds for a DPP with no restrictions (details can be provided for a general case, but this would be out of scope of this note). Thus, all conditions for hyperuniformity of DPPs can be written using, instead of the variance ${\mathbb {V}}\left( \mathcal {X(}\Omega \mathcal {)}\right)$ of ${\mathcal {X}}$, the $L^{1}$ rate of convergence of the associated finite DPP ${\mathcal {X}}^{N_{\Omega }}$, $\int _{{\mathbb {R}}^{2d}}\left| \rho _{1}^{N_{\Omega }}(z)-1_{\Omega }(z)\right| \,dz$.

## Data Availability

All data generated or analysed during this study are included in this published article.

## References

[1] Abreu LD (2010). Sampling and interpolation in Bargmann–Fock spaces of polyanalytic functions. Appl. Comput. Harm. Anal..

[2] Abreu LD, Gröchenig K, Romero JL (2016). On accumulated spectrograms. Trans. Am. Math. Soc..

[3] Abreu, L.D., Pereira, J.M., Romero, J.L., orquato, S.T.: The Weyl–Heisenberg ensemble: hyperuniformity and higher Landau levels. J. Stat. Mech. Theor. Exp. 043103 (2017)

[4] Abreu LD, Pereira JM, Romero JL (2017). Sharp rates of convergence for accumulated spectrograms. Inverse Prob..

[5] Abreu LD, Gröchenig K, Romero JL (2019). Harmonic analysis in phase space and finite Weyl–Heisenberg ensembles. J. Stat. Phys..

[6] Askour, N., Intissar, A., Mouayn, Z.: Espaces de Bargmann g énéralisés et formules explicites pour leurs noyaux reproduisants. C. R. Acad. Sci. Paris Sér. I Math. **325**, 707–712 (1997)

[7] Charles L, Estienne B (2020). Entanglement entropy and Berezin–Toeplitz operators. Commun. Math. Phys..

[8] DeMari F, Feichtinger HG, Nowak K (2002). Uniform eigenvalue estimates for time-frequency localization operators. J. Lond. Math. Soc..

[9] Demni N, Lazag P (2019). The Hyperbolic-type point process. J. Math. Soc. Jpn..

[10] Eisert J, Cramer M, Plenio MB (2010). Area laws for the entanglement entropy—a review. Rev. Mod. Phys..

[11] Estienne B, Stéphan JM, Witczak-Krempa W (2022). Cornering the universal shape of fluctuations. Nat. Commun..

[12] Fenzl M, Lambert G (2022). Precise deviations for disk counting statistics of invariant determinantal processes. Int. Math. Res. Not..

[13] Gautier, G.: On sampling determinantal point processes. Ph.D. thesis. Ecole Centrale de Lille (2020)

[14] Ghosh S, Lebowitz JL (2017). Fluctuations, large deviations and rigidity in hyperuniform systems: a brief survey. Indian J. Pure Appl. Math..

[15] Ghosh S, Lebowitz JL (2018). Generalized stealthy hyperuniform processes: maximal rigidity and the bounded holes conjecture. Commun. Math. Phys..

[16] Gioev D, Klich I (2006). Entanglement Entropy of Fermions in any dimension and the Widom conjecture. Phys. Rev. Lett..

[17] Gröchenig, K.: Private communication

[18] Gröchenig K (2001). Foundations of Time-Frequency Analysis.

[19] Haimi A, Hedenmalm H (2013). The polyanalytic Ginibre ensembles. J. Stat. Phys..

[20] Hough, J.B., Krishnapur, M., Peres, Y., Virá g, B.: Zeros of Gaussian analytic functions and determinantal point processes, volume 51 of University Lecture Series. American Mathematical Society, Providence, RI (2009)

[21] Katori, M., Shirai, T.: Partial isometries, duality, and determinantal point processes. Random Matrices Theory Appl. 2250025 (2021)

[22] Lacroix-A-Chez-Toine B, Majumdar SN, Schehr G (2019). Rotating trapped fermions in two dimensions and the complex Ginibre ensemble: exact results for the entanglement entropy and number variance. Phys. Rev. A.

[23] Marceca, F., Romero, J.L.: Spectral deviation of concentration operators for the short-time Fourier transform. arXiv:2104.06150 (2021), Studia Math. (2023) 10.4064/sm220214-17-10

[24] Matsui T, Katori M, Shirai T (2021). Local number variances and hyperuniformity of the Heisenberg family of determinantal point processes. J. Phys. A Math. Theor..

[25] Shirai T (2015). Ginibre-type point processes and their asymptotic behavior. J. Math. Soc. Jpn..

[26] Torquato S (2018). Hyperuniform states of matter. Phys. Rep..

[27] Torquato S, Stillinger FH (2003). Local density fluctuations, hyperuniform systems, and order metrics. Phys. Rev. E.

[28] Torquato, S., Scardicchio, A., Zachary, C.E.: Point processes in arbitrary dimension from Fermionic gases, random matrix theory, and number theory. J. Stat. Mech. Theory Exp. 11019 (2008)

